# Increase of genetic diversity indicates ecological opportunities in recurrent-fire landscapes for wall lizards

**DOI:** 10.1038/s41598-019-41729-6

**Published:** 2019-03-29

**Authors:** Diana Ferreira, Catarina Pinho, José Carlos Brito, Xavier Santos

**Affiliations:** 1 CIBIO, Centro de Investigação em Biodiversidade e Recursos Genéticos da Universidade do Porto, InBIO, Laboratorio Associado. R. Padre Armando Quintas, 4485-661 Vairão, Portugal; 2 Departamento de Biologia da Faculdade de Ciências da Universidade do Porto. Rua Campo Alegre, 4169-007 Porto, Portugal

## Abstract

Socioeconomic and climatic factors are modifying fire regimes with an increase of fire frequency and extension. Unfortunately, the effects of recurrent fires on biological processes that ultimately affect the genetic diversity of animal populations are mostly unknown. We examined genetic patterns of diversity in the wall lizard *Podarcis guadarramae* in northern Portugal, one of the European regions with the highest percentage of burnt land. This species is a small saxicolous lizard as it inhabits natural outcrops and artificial stone walls, likely in recurrent-fire landscapes. We genotyped nine microsatellites from ten populations selected according to a gradient in fire recurrence, and compared genetic diversity indexes and demographic patterns among them. At the population level, we hypothesize that a high level of mortality and population bottlenecks are expected to reduce genetic heterozygosity in sampled localities affected by recurrent fires. Alternatively, genetic signatures are expected to be absent whether fire did not cause high mortality. Regardless of levels of mortality, we expect a gain in genetic diversity whether recurrent fires facilitate lizard dispersal and migration due to the increased quality of the habitat for wall lizards. At the regional level, we examine whether a recurrent fire regime may disrupt the spatial structure of populations. Our results showed an increase in genetic diversity in recurrently burnt populations, and a decline in longer-unburnt populations. We did not detect bottleneck effects in repeatedly-burnt populations. High genetic diversity in recurrent fire populations suggests a high dispersion rate between adjacent metapopulations and perhaps immigration from outside the fire boundary. At the regional level, lizard populations show low differentiation and weak genetic structure, suggesting no effects of fire. This study confirms field-based censuses showing that recurrent-fire regimes give ecological opportunities to wall lizards that benefit from habitat openness.

## Introduction

Wildfires have played a determining role in shaping the evolution and function of many ecosystems around the world^1,2^. More than a local ecological disturbance, fire is a global ecosystem process^2^, with approximately one third of the land mass experiencing intensive burning^3^. In the last decades, fire-prone regions as the western Mediterranean are experiencing a major shift in the fire regime with an increase of fire frequency and extension^4^. The main driver of this shift is socioeconomic, i.e. increased amount of fuel due to rural abandonment^4,5^, in parallel to a global climate warming^6,7^. Shifts in particular fire-regimes can have devastating impacts on the sustainability of many ecosystem components^8^.

Fire shapes community composition for many taxonomic groups especially due to its major effect on the habitat structure^9^. Understanding the emergent genetic patterns of diversity resulting from habitat disturbance is critical in predicting how species will adapt to and persist in changing environments. However, the role of fire-regime as a driver of the patterns and distribution of genetic diversity is poorly understood^10,11^. Due to their mobility, the response of animals to fire is complex^12^, and the lack of information about the genetic patterns resulting from responses of organisms to fire is especially relevant^13^. To understand this issue, one must consider that the demographic impact of fire may depend on particular life history traits, such as dispersal ability, environmental requirements^14^, and resistance to fire^15^. The complexity builds up when both mortality and recruitment interact to determine the consequences of fire on within-population genetic diversity^11^. On the one hand, high mortality translates into population bottleneck, thereby reducing effective population sizes and producing a random loss of genetic diversity^16^. On the other hand, post-fire population recovery can stem from either survivors or through colonizers from adjacent unburnt areas^17^, which may also have an impact on the distribution patterns of genetic diversity. The loss of allelic diversity will be low if there is either a high survival rate^18^ or if recruitment comes from multiple sources^19,20^. Post-fire population dynamics, such as genetic drift and/or migration, can affect genetic differentiation between populations^21,22^. Conversely, if post-fire migration is facilitated between populations, increased gene flow can reduce genetic differentiation^23,24^. This is the case in open-habitat or early colonizer species, which increase their local density and genetic variation in recently burnt sites^19,20^. In contrast, genetic diversity in long-unburnt specialists may decrease due to habitat fragmentation patterns imposed by fire^25^.

In this study, we have investigated the effects of a recurrent fire regime on the genetic diversity and spatial structure of the wall lizard *Podarcis guadarramae*^26^ in northern Portugal. Wildfire is one of the most important agents of landscape change in Portugal^27^. The study area, in particular, is one of the European regions with the highest percentage of burnt land due to a combination of socioeconomic (continuous surface of highly flammable eucalyptus and pine forests) and climate (high rainfall rate episodes followed by dry periods) factors. *Podarcis guadarramae* is a small-size saxicolous wall lizard^28^ that uses rocks, trunks, and bare ground for thermoregulation, foraging and shelter^29^. In northern Portugal, this lizard has a patchy distribution with populations located in natural open rocky outcrops and artificial stone walls surrounding agriculture fields^26^. The forested and bush matrix isolates lizard populations and hampers inter-population contact due to the low dispersion ability of this species through vegetated areas. Wildfire reduces vegetation cover, increases the extent of open outcrops, and allows lizard dispersion among patches with favourable (open) habitats. These life-history traits can explain the positive population responses of *P. guadarramae* to fire observed in northern Portugal^30,31^. However, the demographic processes (e.g. mortality, survival, migration) underlying the positive response of wall lizards to fire are unknown. For this reason, we have collected lizard samples in ten populations selected according to a gradient of disturbance level (i.e. number of fires and time since the last fire). The objective of this study was to examine how recurrent fire regimes affect: i) at the population level, the genetic diversity of each lizard population sampled, and ii) at the regional level, the geographic/genetic structure of the sampled *P. guadarramae* populations. Emergent genetic patterns of diversity can help to understand demographic processes linked to fire recurrence and habitat use. Based on *P. guadarramae* life-history traits, we have addressed the following hypotheses.

At the population level, if fire causes high mortality rate, wall lizard populations located on rocky outcrops that have experienced higher number of fires are expected to exhibit genetic signatures consistent with recent demographic bottlenecks and low genetic diversity. Alternatively, such genetic signatures are expected to be absent (i.e. no diversity loss) when fire does not cause severe mortality. Moreover, if recurrent fires improve habitat quality and facilitate lizard dispersal and migration, genetic diversity gains are expected regardless of lizard mortality caused by fire.

At the regional level, we hypothesize that fire can shape population structure due to the expected fire impacts over the genetic diversity and ultimately over population differentiation. Accordingly, we expect fire to disrupt the spatial structure of populations by decreasing the naturally expected correlation between genetic and geographic distances.

## Results

From the initial battery of nine microsatellite loci genotyped, one (Ph17) showed presence of null alleles in seven out of 10 populations (Supplementary material Table S1) and for this reason it was excluded from further analyses. Five out of the remaining eight loci showed evidence for the presence of null alleles and/or stuttering (Supplementary material Table S1); however, these patterns affected only a minority of populations. Despite the existence of null alleles, no deviations from either HWE or LE were found in the eight loci, and therefore we opted not to exclude any other loci.

Mean number of alleles per locus was 7.73 (±0.67 s.d.; range 5–19 alleles). Overall, the expected heterozygosity (H_E_) of the eight loci showed moderate values, ranging from 0.663 to 0.732 (average 0.694 ± 0.019), while the observed heterozygosity averaged 0.647 (±0.026). The allelic richness (A_R_) per population ranged from 5.922 to 7.763, whereas the mean number of alleles (N_A_) per population varied from 6.125 to 8.625 (Table 1). Four of the populations sampled had private alleles, especially those populations subjected to recurrent fire-regimes (Table 1). A_R_, N_A_ and H_E_ diversity indices showed a general pattern of increasing diversity values from unburnt towards burnt populations, with significant differences detected for three out of five pairs, suggesting that fire changed the pattern of genetic diversity. When pools of populations with the similar condition (unburnt vs. repeatedly burnt) were compared, a clear difference was reported for N_A_ and P_A_ (Table 1). Similarly, there was a trend of increasing F_IS_ towards the repeatedly burnt populations detected for all pairs, although pairwise differences within each pair was only significant for one population (Table 1).

**Table 1:** Summary of the genetic diversity indices calculated for each of the ten *Podarcis guadarramae* populations sampled, and P values of the comparisons between pairs of populations at each location in N_A_, P_A_1, and H_E_. The same comparisons were performed for the pool of unburnt (UN) populations against burnt (BU) populations.

Population	N	N_A_	P	A_R_	P_A_2	P_A_1	P	H_O_	H_E_	P	F_IS_
Leonte UN	21	7.125	0.367	6.786	—	8	0.376	0.718	0.703	0.757	0.004
Leonte BU	20	7.125		6.736	—	8		0.608	0.686		0.139
Lindoso UN	20	6.750	**0.047**	6.474	—	9	**0.047**	0.619	0.677	0.440	0.112
Lindoso BU	17	7.250		7.152	—	13		0.621	0.677		0.114
Santo Tirso UN	20	7.375	0.234	6.961	—	8	0.234	0.659	0.719	0.280	0.109
Santo Tirso BU	21	7.750		7.327	1	11		0.648	0.732		0.140
Moledo UN	20	6.875	**0.001**	6.507	1	6	**0.001**	0.665	0.672	0.220	0.036
Moledo BU	22	8.625		7.763	3	20		0.669	0.693		0.057
Póvoa de Lanhoso UN	20	6.125	**0.005**	5.922	—	11	**0.005**	0.656	0.663	**0.030**	0.036
Póvoa de Lanhoso BU	20	7.750		7.251	5	24		0.613	0.712		0.170
UN populations		10.000	**<0.0001**			2	**<0.0001**		0.725	0.110	
BU populations		11.750				16			0.738		

N, number of individuals sampled; N_A_, mean number of alleles; A_R_, allelic richness; P_A_1, number of private alleles within each pair; P_A_2, number of private alleles for each population relative to the total sample; H_O_, observed heterozygosity; H_E_, expected heterozygosity; F_IS_, deviation from HW proportions.

**Figure 1:**
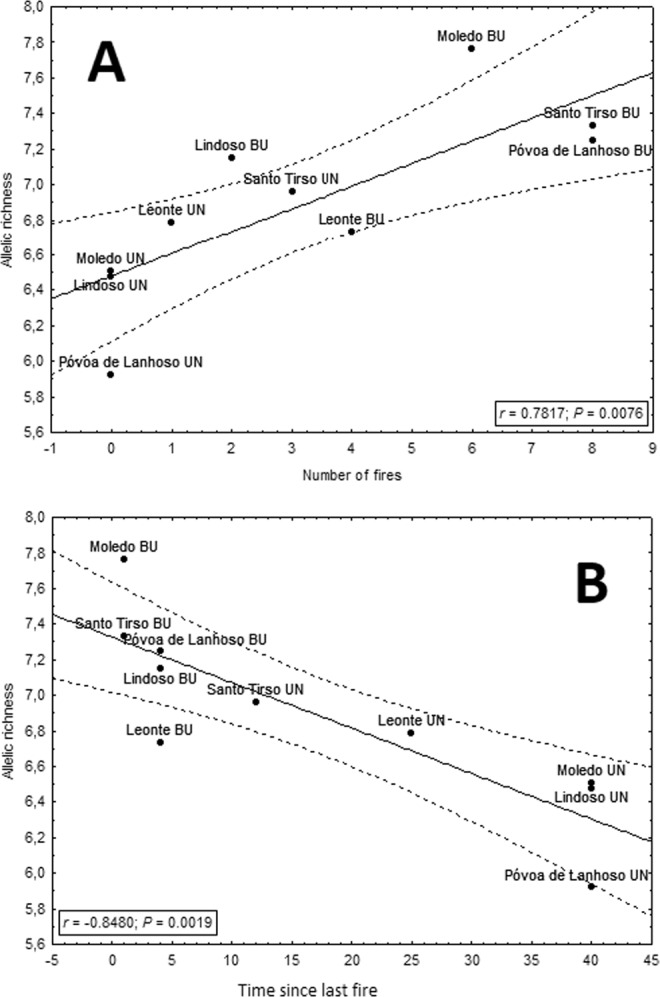
Variation of allelic richness with the number of fires (**A**) and the time since fire, TSLF (**B**) that each population of *Podarcis guadarramae* experienced from 1975–2014. UN: unburnt, and BU: burnt populations.

All genetic diversity measures (A_R_, N_A_, H_E)_) and F_IS_ were positively correlated with the number of fires that occurred at each population (Fig. 1A for A_R_, and Supplementary material Fig. S2 for remaining metrics). Moreover, these measures were negatively correlated with the time since the last fire occurred at each population (Fig. 1B for A_R_, and Supplementary material Fig. S3 for the rest of the metrics).

Almost all population pairs showed significant, although low, allele-frequency-based differentiation (Supplementary material Table S4). Significant pairwise F_ST_ values were low (0.015–0.088). With exception of Moledo pair, all remaining locations showed no significant differentiation regarding the pairs of populations. AMOVA results revealed that all levels of genetic differentiation were significant (Table 2). Most of the genetic variation was found within populations (96.2%); only a small but significant percentage was attributed to the variation among the five population pairs (2.17%), and to between pairs of populations within each pair (1.63%), which corroborates the fact that almost all pairwise F_ST_ values were significant.

**Table 2:** Summary results of analysis of molecular variance (AMOVA) within and among locations of *Podarcis guadarramae* sampled.

Source	SS	VC	Variation (%)	Fixation indices	
Among location	39.429	0.06429	2.17087	F_ST_	0.03796^**^
Among populations within location	23.851	0.04812	1.62500	F_SC_	0.01661^*^
Within populations	1106.559	2.84894	96.20413	F_CT_	0.02171^**^
Total	1169.839	2.96135			

SS – Sum of squares; VC – Variance component; ^*^P = 0.003; ^**^P = 0.000.

**Figure 2:**
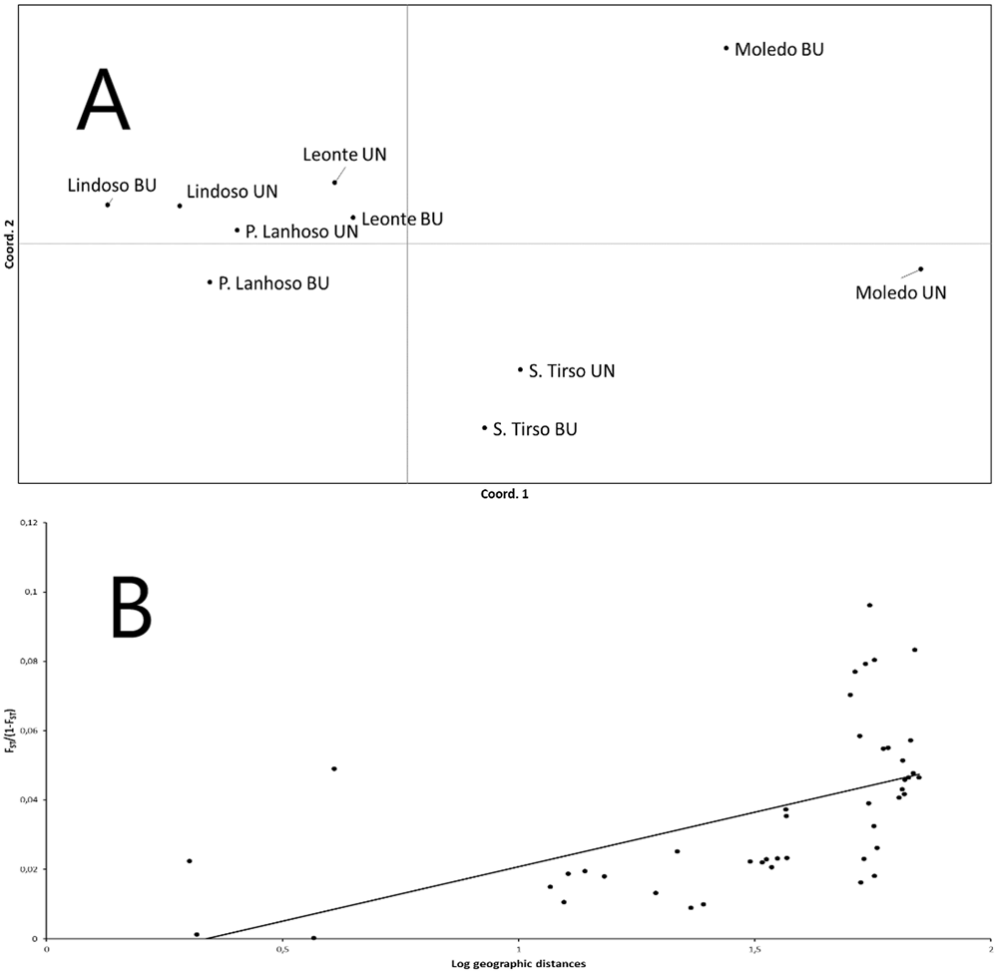
Patterns of genetic variation plotted with a Principal Coordinate Analysis based on Slatkin’s linearized F_ST_ (F_ST_/(1 − F_ST_)) genetic distance between all the ten populations sampled (**A**), and relationship between F_ST_ (F_ST_/(1 − F_ST_)) genetic distance and log-transformed geographic distances (in km) between populations (**B**).

The Mantel test showed a significant correlation between genetic distance (Supplementary material Table S5) and log-transformed geographical distance (r_M_ = 0.547; P < 0.0001; 999 permutations; Fig. 2B), meaning that the observed differences were most likely a result of spatial structure and isolation by distance and not fire history. This result can be visualized in the PCoA (the two coordinate axes together explained 71.98% of the total genetic variation) since it mirrors the paired organized spatial location of sampled populations (Fig. 2).

Bottleneck test results were not robust to changes in the assumptions regarding mutation models (Table 3). M-ratio values were generally high across populations, suggesting low general evidence for bottlenecks. Nevertheless, there were five localities (notably including both unburnt and burnt populations) in which M-ratios fall below the highest *Mc* threshold which corresponds to the assumptions of the strict SMM (which is unlikely to fit most microsatellites) and 4Neµ = 5 (see Supplementary material Table S6). Only two unburnt populations (Leonte and Póvoa de Lanhoso) showed M-ratios lower than the *Mc* values calculated relaxing the assumptions of the strict SMM, but these values are still quite high and hence not robust to small changes in model assumptions. With respect to the heterozygosity excess test, results ranged from significant heterozygosity excess in all populations (when assuming the IAM) to no evidence of bottleneck in any population (when assuming the SMM). When fitting the TPM, the evidence varied along the proportion of single step mutations included in the model. Assuming the default parameters incorporated in BOTTLENECK (70% of mutations fitting the SMM, with a variance of 30%), only two unburnt localities showed significant heterozygote excess, as suggested by the Wilcoxon test. In summary, comparing the various tests, only the unburnt population located at Leonte showed consistent evidence for the occurrence of a bottleneck. Even though results are not robust to changes in the mutation model, it is clear that the evidence is not larger for bottlenecks in repeatedly burnt, when compared to unburnt, localities.

**Table 3:** Bottleneck test results. The M-ratio was calculated after Garza & Williamson (2001), and the heterozygosity excess test was performed after Cornuet & Luikart^77^ for the infinite allele model (IAM), strict stepwise mutation model (SMM) and two-phase mutation model (TPM) with different proportions of SMM-type mutations (0%, 50%, 90%) and variance (5%, 30%, 36%).

Locality	M-ratio	heterozygosity excess						
		IAM	TPM					SMM
			0–36%	50–36%	90–36%	70–30%	75–5%	
Leonte UN	**0.786**	**	**	**	*	*	n.s.	n.s.
Leonte BU	0.858	*	*	n.s.	n.s.	n.s.	n.s.	n.s.
Lindoso UN	**0.821**	*	*	n.s.	n.s.	n.s.	n.s.	n.s.
Lindoso BU	0.83	*	*	n.s.	n.s.	n.s.	n.s.	n.s.
Santo Tirso UN	0.842	*	*	*	n.s.	*	n.s.	n.s.
Santo Tirso BU	**0.802**	*	*	*	n.s.	n.s.	n.s.	n.s.
Moledo UN	0.832	*	*	*	n.s.	n.s.	n.s.	n.s.
Moledo BU	**0.808**	*	n.s.	n.s.	n.s.	n.s.	n.s.	n.s.
Póvoa do Lanhoso UN	**0.746**	*	*	n.s.	n.s.	n.s.	n.s.	n.s.
Póvoa do Lanhoso BU	**0.822**	**	**	*	n.s.	n.s.	n.s.	n.s.

Values of the M-ratio lower than the highest critical M (*Mc*) value estimated for a range of parameter values appropriate for our data (0.8298, for the strict SMM case and 4Neµ = 5) are shown in bold. The significance of the heterozygosity test, calculated based on a Wilcoxon test, is shown as follows: ^**^0.001 < p < 0.01; ^*^0.01 < p < 0.05; n.s., not significant.

## Discussion

Our study design (a gradient of recurrent-fire histories) provided the opportunity to perform tests to enlighten which genetic patterns and processes are driven by the occurrence of recurrent wildfires, and to extend the knowledge in this research field that is currently lacking information^11,13^. We demonstrated emergent genetic patterns of diversity resulting from demographic responses to fire by the wall lizard *P. guadarramae* at population level (i. e. genetic diversity indexes), but not at regional level (i. e. relationship between genetic and geographic distances). Due to its environmental requirements of open outcrops^32^, *P. guadarramae* benefits from recurrent burned landscapes dominated by granitic rocks where lizard populations achieve high effective population sizes^30,33^. The positive response of *P. guadarramae* to fire in northern Portugal^30,31^ showed that recurrent-burnt landscapes give ecological opportunities to this wall lizard that seem to be related with the increase of genetic variability in the repeatedly burnt populations.

### Wildfires alter the patterns of genetic diversity in *P. guadarramae*

Population recovery following major disturbances such as wildfires is likely a reflection of two non-mutually exclusive biological processes: i) abundance of survivors; and ii) post-fire recruitment (e.g. local reproduction or immigration)^13,16,17^. Some studies have documented a short-term post-fire decline of fire-sensitive species^34^, although studies on vertebrates have shown that fires rarely cause complete mortality and that residual populations may remain *in situ* in burnt areas^16,18^, specifically cliff- and rock-specialist species for which habitat is not destroyed by fire^34^. Biological processes, such as mortality, survival, and post-fire recolonization can affect genetic diversity in contrasting ways:^11^ reductions in heterozygosity and allelic richness resulting from population bottlenecks^22^; increases in genetic diversity and reduced regional genetic differentiation resulting from high immigration rates^19,20^; and intermediate values of unchanged genetic diversity when mortality is low or is compensated by immigration^18^.

Analyses of genetic diversity helped us to unravel how recurrent fires affect the population dynamics of *P. guadarramae*. Our results clearly showed that fires altered the genetic diversity of wall lizards. Our first expectation was that fires could alter patterns of genetic diversity in burnt populations by decreasing it through successive bottlenecks and founder effects. These results have been found in populations of butterflies^25^ and lizards^23^ and have been further attributed to bottlenecks in several taxa such as mammals^35^, birds^22^, and lizards^36^. Consequently, burnt populations would become more differentiated from unburnt populations by means of genetic drift. However, our results did not meet this expectation. Interestingly, we obtained the opposite pattern: significantly increasing allelic diversity with number of fires, and conversely, a significant decreasing of allelic diversity with time since the last fire. This trend was valid for the overall correlation between fire and genetic diversity parameters, and also, in general, to local pairwise comparisons.

We suggest that the pattern of higher genetic diversity (mean number of alleles, allelic richness, private alleles, expected heterozygosity) found in repeatedly burnt populations of *P. guadarramae* can be explained by a combination of both high survival rate, mobility, immigration, and a post-fire increase in the carrying capacity of disturbed habitats.

The likelihood of immediate survival of an individual during a fire will be influenced by the severity of the fire, its distance to potential refuges and behavioural mechanisms the organisms may use to avoid direct flames and heat^16,37^. Refuges enhance immediate survival during a fire event but can also facilitate the post-fire persistence of individuals and populations within the burnt landscape by providing resources in the short- (food, shelter) or long-term (resident habitat) from a series of disjunct isolated populations to a metapopulation, and to a patchy population linked by frequent movements^16,17,38,39^.

The response of *P. guadarramae* to fire has been demonstrated in several demographic studies in the region^30,31^. Physiological experiments^40,41^ and habitat preference studies have highlighted that this lizard copes very well with post-fire habitat conditions. These studies and our personal observations in recently burnt spots support the increased dispersal ability of individuals in post-fire habitats that brings new diversity (and increase allelic richness and heterozygosity). Increased dispersal activity in recently burnt habitats was the cause of increased genetic diversity in the agamid *Amphibolurus norrisi* in Australia^42^. Schrey, Ashton, *et al*. (2011) also found increased genetic diversity in burnt areas, in one of the examined lizard species. They tested a metapopulation source/sink model based on the habitat preferences of each species, by examining the direction of gene flow (i.e. immigration into or out of preferred habitat). The burnt area indeed received influx of individuals from open-habitat species, increasing genetic diversity. In other taxonomic groups with higher dispersal capability, a post-fire increase in genetic diversity was also reported; e.g. the mountain brushtail possum *Trichosurus cunnunghami*^43^, and the Gran Canaria chaffinch *Fringilla teydea polatzeki*^18^. A combination of life history traits (e.g. dispersal abilities), and habitat characteristics (fire-adapted vegetation, use of unaffected refuges by fire) could have contributed to increase genetic diversity.

Our results suggest that refuges are preponderant both in the survival rate of wall lizards as well as for colonizers. *Podarcis guadarramae* is a highly saxicolous lizard and it is associated with rocky habitats^28,44^. Likewise, its preference for these habitats with a naturally high number of crevices and burrows, where the lizard could take refuge during the fire events, may allow a considerable level of survival among animals (see a similar result for the saxicolous gecko *Tarentola mauritanica*^34^), allowing the retention of a large fraction of the pre-fire genetic diversity. This fact is in line with the lack of evidence for severe bottlenecks in burnt populations.

Moreover, given that fire clears vegetation and exposes previously unsuitable rocky outcrops, unoccupied habitats represent a new ecological opportunity for wall lizards. This not only supports an increase in the local effective population size, but also allows the dispersal of individuals between metapopulations, and potentially, recruitment and settlement of migrants from outside the affected area. Both mobility and migration can positively increase genetic diversity at the population level. Our results shown an increase in F_IS_ in recently burnt localities, which may be in line with Wahlund effects caused by recent migrants from different gene pools^45^. We acknowledge that this hypothesis cannot be corroborated by the absence of pre-fire genetic data nor movement data. However, the negative correlation between genetic diversity and the time since the last fire was indirect evidence of the mobility/migration lizard pattern in burnt populations. Indeed, long-unburnt localities showed reduced genetic diversity of lizard populations likely by vegetation re-growth that reduces lizard mobility and isolates small populations in small rocky spots. The incorporation of knowledge of both direct, in particular the demographic and mobility processes^13^, and indirect effects (shift in habitat structure) of fire is important to understand the underlying processes behind the patterns of genetic diversity found^11^.

### Wildfires did not alter the patterns of geographic differentiation in *P. guadarramae*

The observed general increments of genetic differentiation according to geographic distance (isolation by distance) and not to fire history, suggested limited gene flow among all *P. guadarramae* populations. This result configures a classical metapopulation scenario, especially when individuals show low dispersal ability, like the lizard case^46^, and matches findings from closely related species (*P. bocagei* and *P. carbonelli*) also inhabiting western Iberia, in which, similarly low or slightly higher levels of population differentiation were reported across their distribution areas, yet with a clear correspondence to geography (Pinho, Harris, & Ferrand^47^; Pinho, Kaliontzopoulou, Harris, & Ferrand^48^).

Reptiles show strong succession responses to fire^49^ that are driven by habitat structure changes and are a product of species life history (Santos *et al*.^34^; Smith^50^). Thus, as general rule, the open-habitat dwelling species are favoured by post-fire habitat conditions, then, while the ecological succession is recovering, forest-species increase in abundance whereas open-habitat species tend to decrease (Santos *et al*.^34^; Valentine, Reaveley, Johnson, Fisher, & Wilson^51^). In our study areas (i.e. locations), these metapopulations could be spatially arranged according to the distribution of rocky and agricultural (walls) areas given its saxicolous ecology^28^.

### Concluding remarks

Due to their particular life-history traits (small size, low mobility, ectothermy, low reproductive recruitment), reptiles are important organisms to examine in their responses to fire at different complementary approach levels, i. e. demographic, genetic, ecologic. Given the saxicolous preferences of *P. guadarramae*, all the population trends suggest that this lizard benefits from recurrent fires. Microhabitat preference and post-fire dispersal seem to be traits that help this lizard to resist fire and persist afterwards. Mobility is an attribute that makes fauna a challenging group to examine their responses to fire^12^. However, data on this trait needs to be integrated with other biological traits to capture all relevant information in predicting how reptile populations respond to recurrent disturbances^50^. Integrative approaches are necessary to forecast biodiversity patterns in future scenarios of shifts in fire regime.

## Methods

### Study species: the wall lizard *Podarcis guadarramae*

**Figure 3:**
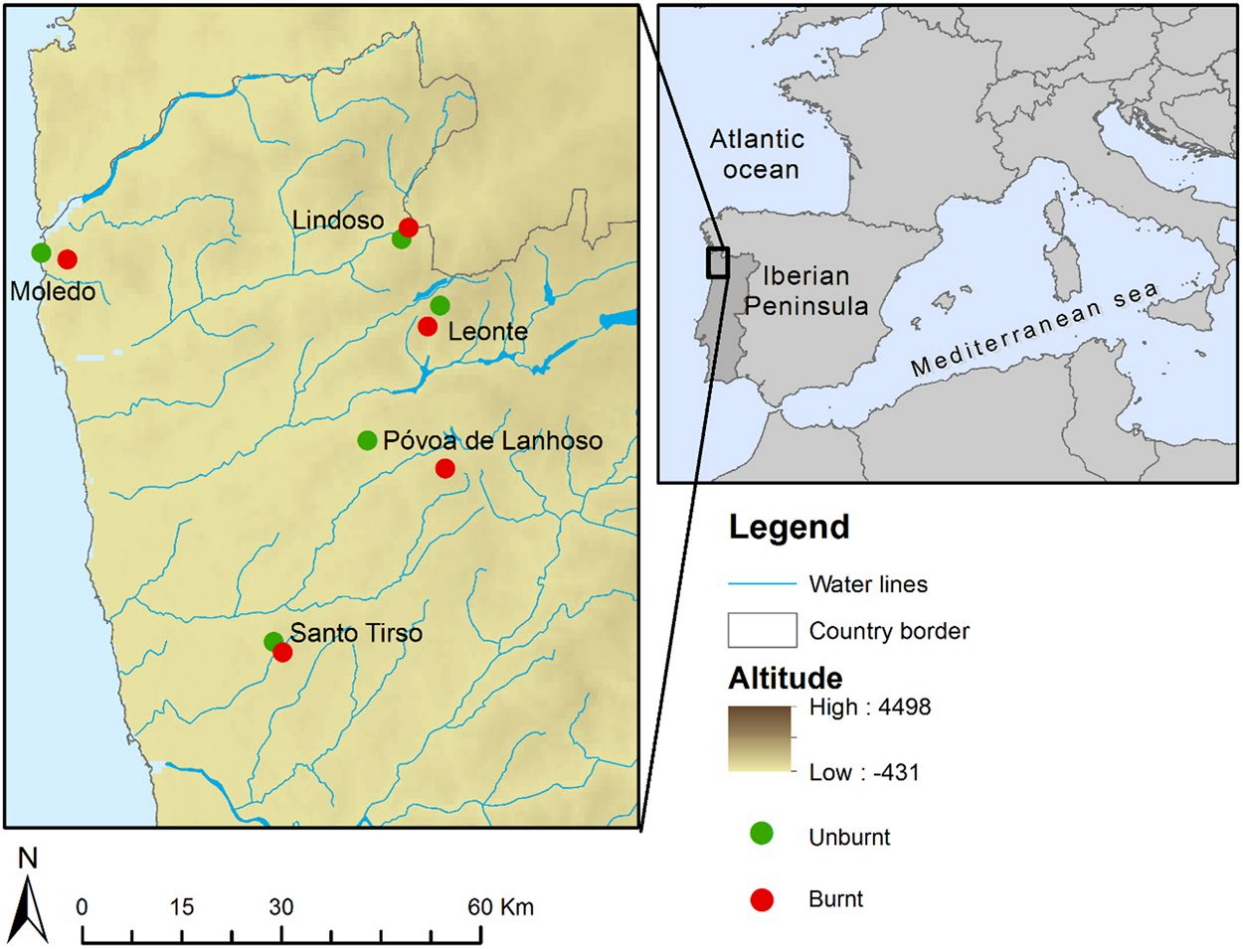
The study area is located in the north-western extreme of Portugal which is the area most affected by fires in Europe. Each location sampled is formed by two populations of Podarcis guadarramae with opposite conditions, one unburnt (green circles) and one burnt (red circles).

Wall lizards of the genus *Podarcis* (Squamata: Lacertidae) are among the most conspicuous, abundant and widely distributed reptiles in Europe and North Africa. *Podarcis guadarramae* occurs in northern Portugal, north-western Spain, and Central Iberian Mountains in Spain (Catarina Pinho, Ferrand, & Harris^52^; Fig. 3). The populations that are the object of this study correspond to the north-western subspecies, *P. g. lusitanicus*. It is a saxicolous and small (50–70 mm adult snout-to-vent length) diurnal lacertid lizard^28^ with Atlantic ecological affinities^53^. Its depressed body shape and flattened skull facilitate entrance into narrow, irregular crevices. Individuals aggregate around favourable open areas with rock crevices, artificial walls, and isolated big blocks^33^.

### Fire regime, site selection and experimental design

Portugal is currently one of the European regions with the highest percentage of burnt land^54–56^. Fire frequency and intensity have increased remarkably since the 1960s^57,58^, due to a combination of socioeconomic (i.e. rural abandonment and traditional practices (Moreira *et al*., 2001); plantations of fire-prone tree species such as eucalypt and pines (Fernandes *et al*.^59^; Moreira *et al*., 2001) and environmental factors (hot, dry summers and cool, wet winters; Nunes *et al*., 2005). Coincident with *P. guadarramae* distribution, the northern part of the country has the highest fire frequency and size (Nunes *et al*., 2005; Pereira *et al*., 2006) due to the dominance of fire-prone tree species (maritime pine, *Pinus pinaster*; and *Eucalyptus* spp.) and shrubs (genus *Erica*, *Calluna*, *Ulex* and *Cytisus*; Carmo, Moreira, Casimiro, & Vaz^60^; Scotto *et al*.^61^).

The sampling area (latitudinal range: 41°05′ to 42°15′ N, and longitudinal range: −8°90′ to −7°76′ W) was located in the transition between the Mediterranean and Eurosiberian biogeographic zones close to the Atlantic coast^62^ with a transitional climate from Atlantic (NW) to Mediterranean (NE). Average annual precipitation ranges from approximately 1200 mm at the coast to 2800 mm at the upper elevations in the interior, mostly falling between October and April and with 0–2 rainless months. The average annual temperature is 14.5 °C, mean maximum and minimum temperatures are respectively 25 °C in July and 4 °C in January^63^. Native forests have become very fragmented, and the characteristic species are *Quercus robur*, *Q. pyrenaica*, *Acer pseudoplatanus*, *Pinus pinaster*, *Pyrus cordata*, and *Ilex aquifolium*. The most common type of vegetation are fire-prone scrub communities composed by genus *Erica*, *Calluna*, *Ulex* and *Cytisus*^64^.

To test how the repeated fire regime that characterizes the study area affected genetic variation in the wall lizard *P. guadarramae*, we selected ten locations based on a digital national cartography of burnt areas from 1975 to 2013 (http://www.icnf.pt/portal/florestas/dfci/inc/info-geo). These locations followed a gradient of recurrent fires and time-since-fire periods. Burnt locations had 5.8 fires on average (range 2–8 fires) during the period 1975–2013 (Supplementary material Table S7. Moreover, locations were selected following a paired-population sampling scheme, each pair composed with locations with contrasting fire histories (from unburnt to repeatedly burnt). Pairs of locations were located on average at 4.9 km distance between each (range 2.0–12.5 km per location; Fig. 3). This sampling design avoids spatial pseudo-replication in fire regimes, and minimizes the potential effect of spatial structuring to mask the effect of recurrent fires on the genetic diversity.

Sampling was conducted between July-October 2014. Except one recurrently burnt population, at least 20 individuals were sampled from each population. Individuals were captured using a noose or by hand. Sampling syntopic individuals was avoided as well as sampling juveniles. The tail tip of each individual was collected and stored in 96% ethanol. All individuals were then released back to their collection site. The experiments were performed in accordance with general guidelines and regulations of live vertebrates. The experimental protocol was approved by the Instituto da Conservação da Natureza e das Florestas (ICNF) from the Portuguese Government that provided the permit for sampling lizards in northern Portugal (number 578/2014/CAPT).

### Laboratory procedures

DNA was extracted from tail muscle of 201 *P. guadarramae* lizards using the EasySpin Genomic DNA Tissue Kit (Citomed, Portugal) following the manufacturer’s instructions. Samples were genotyped at nine microsatellite loci chosen based on further multiplex optimization and reports of scoring errors by Ribeiro^65^; Supplementary material Table S8) which modifies those reported in Agostini *et al*.^66^. PCR products were then separated by size on an automatic sequencer ABI 3130xl Genetic Analyser (Applied Biosystems, U.S.A.) with size standard GS500 LIZ (Applied Biosystems, U.S.A.). Allele scoring was performed with GeneMapper v4.1 (Applied Biosystems, U.S.A.) and checked manually.

### Microsatellite data analyses

The final data set included 201 samples genotyped for nine microsatellite *loci*. No individual or locus was excluded based on missing data. We used MICRO-CHECKER v2.2.3^67^ to test for the presence of null alleles or scoring errors. We used ARLEQUIN v3.5^68^ to test for departure from Hardy-Weinberg equilibrium (HWE) and linkage disequilibrium (LD). Bonferroni corrections were applied due to multiple tests^69^. With the final data set, we performed three types of analyses: i) population genetics; ii) population differentiation; and iii) bottleneck tests.

### Analysis of genetic diversity

Using Arlequin, we calculated allele frequencies, expected (H_E_) and observed (H_O_) heterozygosities, mean number of alleles (N_A_), and number of private alleles (P_A_) for each population. We also calculated allelic richness (A_R_; calculated using a rarefaction methodology considering the lowest number of individuals genotyped in a locality sensu El Mousadik & Petit^70^) and inbreeding coefficient F_IS_^71^ with FSTAT v2.9.3.2^72^.

Differences in genetic diversity indexes (N_A_, P_A_, H_E_, and F_IS_) between each population pair, and between burnt and unburnt populations as a whole were explored using a permutation approach with 999 permutations. These analyses were performed with Python 2.7 scripts (available from the authors upon request).

We further explored the relationship between genetic diversity indices (N_A_, A_R_, H_E_, F_IS_) and fire history variables (number of fires, time since the last fire) by using simple linear regression in STATISTICA v8.0^73^. Due to the independence of each fire event, these analyses disregard the paired sampling design and focus on an overall comparison of fire-history and genetic diversity among all ten populations sampled.

### Analysis of population differentiation

To test for genetic differentiation among all populations pairs, pairwise F_ST_ values were calculated. A hierarchical population structure was evaluated through an analysis of molecular variance (AMOVA) grouping by pairs of location in order to test the null hypothesis that genetic variation was not associated with spatial structure. Both analyses were performed in ARLEQUIN using 10,000 permutations.

Geographic distances between populations were calculated from the pairwise Euclidean distance according to the latitude and longitude of each location with Geographic Distance Matrix Generator, version 1.2.3 (Ersts, American Museum of Natural History, Center for Biodiversity and Conservation, http://biodiversityinformatics.amnh.org/open_source/gdmg). We tested for isolation by distance by regressing log transformed geographic distances (in km) against Slatkin’s linearized F_ST_ (F_ST_/(1 − F_ST_)). Statistical significance was examined by Mantel test with 999 permutations in PASSaGE^74^. A Principal Coordinate Analysis (PCoA) was computed in GenAlex 6.5^75,76^ to visualize patterns of genetic differentiation among populations.

### Bottleneck tests

Bottlenecks (such as those expected to derive from the occurrence of wildfires) can be detected by taking into account patterns of microsatellite allele size distribution and/or frequency data^77–79^. To evaluate the hypothesis that wildfires caused a significant reduction in population sizes in lizard populations, we employed two commonly used methods to detect population bottlenecks: Garza and Williamson’s (2001) M- ratio, and a test for heterozygosity excess developed by Cornuet & Luikart^77^. This test is based on the principle that, when populations experience a sudden decrease in population size, the number of alleles decreases faster than heterozygosity. Hence, a transient excess in heterozygosity can be detected in bottlenecked populations when compared to mutation-drift equilibrium expectations. We used the software BOTTLENECK v. 1.2^80^ to evaluate such heterozygosity excess. Because the expected patterns of variation vary extensively depending on the mutation model assumed, the test of the robustness of the inferences was performed under the infinite allele model (IAM, Kimura & Crow^81^), the strict stepwise mutation model (SMM, Ohta & Kimura^82^), and under a wide range of two-phase mutation model (TPM, Di Rienzo *et al*.^83^) parameters. BOTTLENECK provided several tools to compare hypothesis; we chose the Wilcoxon’s test, since it has been shown to be the most powerful, especially for data sets with a small number of loci such as in our case^80^.

The M-ratio test quantifies gaps in the distribution of microsatellite allele sizes. Under a bottleneck, the number of alleles *k* is expected to decrease faster than allele range *r*. Values of M = *k/r* lower than expected given equilibrium expectations may thus be indicative of a bottleneck. We used the R package strataG^84^ to perform the calculation of M and the script “Critical_M” (obtained at https://swfsc.noaa.gov/textblock.aspx?Division=FED&id=3298) was used to determine the “Critical M” (*Mc*) value. This program calculates the M-ratio from simulated data sets under equilibrium for a given set of relevant parameter values and returns the value representing the fifth percentile of this distribution. We calculated *Mc* based on the sample size and the number of loci specific to our study. We also used a range of reasonable parameter values for the pre-bottleneck population mutation rate (from 5 to 20) and for the microsatellite mutation model parameters, including both the strict SMM as well as allowing for larger size mutations in varying proportions (see Supplementary material for details).

Both types of tests rely up to a great extent on the mutation model assumed for microsatellite loci. Except in the case of the IAM, the mutation models typically used for microsatellite data do not tolerate the existence of alleles resulting from insertions and deletions in the flanking regions. An observation of allele sizes in our case revealed the existence of a few alleles not compatible with strict repeat-number mutations. Therefore, to accommodate our data to the models under which these hypotheses were tested, we performed the analyses described above in a reduced data set resulting from the removal of the loci that, in each population, deviated from the expected repeat number pattern. This resulted in data sets containing six loci in all localities, except Santo Tirso – burnt, where five loci were retained.

## Supplementary information

Supplementary information

Dataset 1

## Data Availability

All data generated or analysed during this study are included in this published article (and its Supplementary Information files).
