# Supplementary material for: Increase of genetic diversity indicates ecological opportunities in recurrent-fire landscapes for wall lizards
Source: Sci Rep. 2019 Mar 29;9:5383. doi: 10.1038/s41598-019-41729-6 (PMC6441018; doi:10.1038/s41598-019-41729-6)
Supplement: Supplementary file 1 — Supplementary information [file 41598_2019_41729_MOESM1_ESM.docx]

**Increase of genetic diversity indicates ecological opportunities in recurrent-fire landscapes for wall lizards**

**Diana Ferreira, Catarina Pinho, José Carlos Brito, Xavier Santos**

**Supplementary information**

**Table S1.** Presence of null alleles (NA) and stuttering of the genotyped loci in each population of *P. guadarramae*. Locus Ph17 was eliminated.

|  | Leonte | | Lindoso | | S. Tirso | | Moledo | | P. Lanhoso | |
| --- | --- | --- | --- | --- | --- | --- | --- | --- | --- | --- |
|  | UN | BU | UN | BU | UN | BU | UN | BU | UN | BU |
| Ph17 | NA |  |  |  | NA | NA | NA | NA | NA | NA |
| Ph21 |  |  |  |  |  |  |  |  |  |  |
| Ph30 |  | NA | NA |  | NA |  |  |  | NA |  |
| Ph38 |  |  |  |  |  |  |  |  | NA |  |
| Ph50 |  |  |  | NA |  |  |  |  |  |  |
| Ph43 |  | NA |  |  |  | NA |  |  |  |  |
| Ph70 |  |  |  |  |  |  |  |  |  |  |
| Ph81 |  |  |  |  |  |  |  |  |  |  |
| Ph128 |  | NA | NA  Stutter | NA  Stutter |  |  |  |  | NA  Stutter |  |

**Figure S2.** Variation of the expected heterozygosity (A), F_IS_ values (B), and mean number of alleles (C), with the number of fires that each population of P. guadarramae experienced since 1975. r – Linear correlation coefficient between variables. UN: unburnt population; BU: burnt population


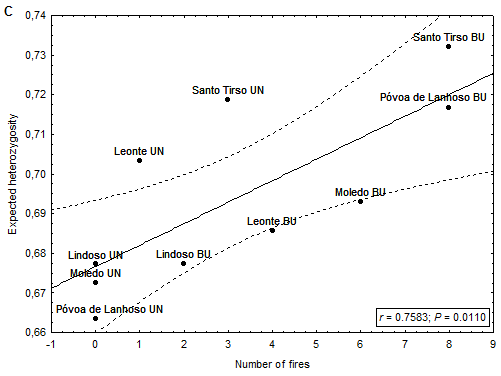

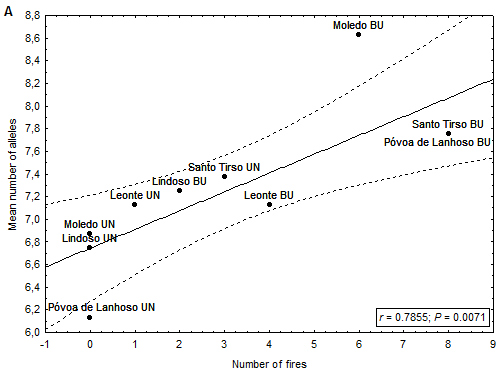

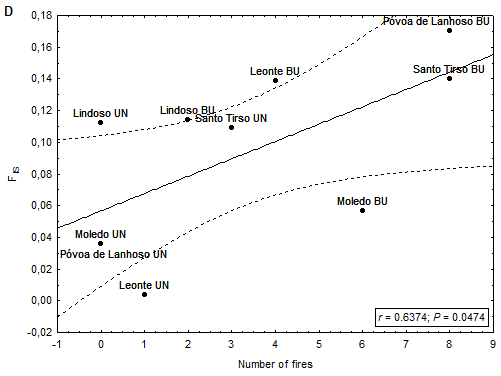


**(A)**

**(B)**

**(C)**

**Figure S3.** Variation of the mean number of alleles (A), expected heterozygosity (B), and F_IS_ values (C), with the time since last fire (TSLF, in years) that each population of P. guadarramae experienced since 1975. r – Linear correlation coefficient between variables. UN: unburnt population; BU: burnt population.


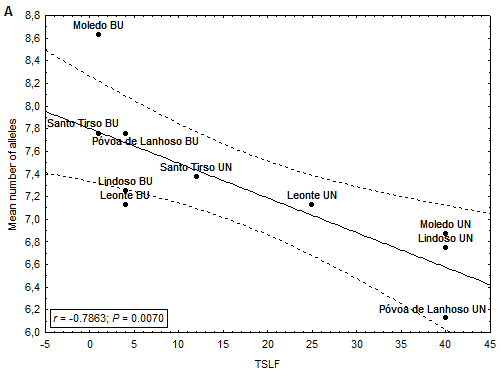

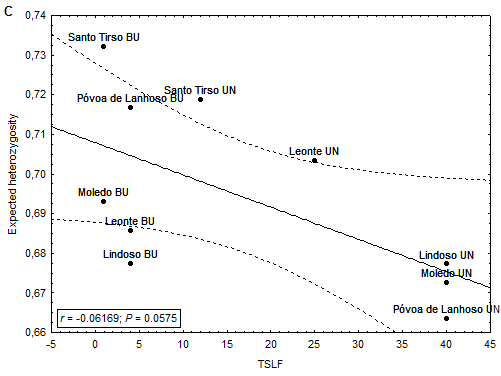

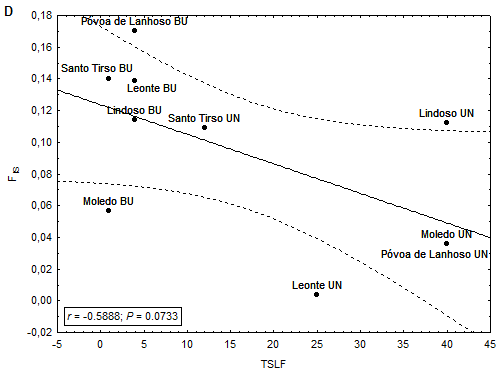


**(A)**

**(B)**

**(C)**

**Table S4.** Measures of pairwise differentiation for ten populations of P. guadarramae sampled based on F_ST_ (below the diagonal; bold values are significant) and significance values with standard deviation between brackets (above diagonal). UN – unburnt; BU – burnt.

|  | **Leonte UN** | **Leonte BU** | **Lindoso UN** | **Lindoso BU** | **S. Tirso UN** | **S. Tirso BU** | **Moledo UN** | **Moledo BU** | **P. Lanhoso UN** | **P. Lanhoso BU** |
| --- | --- | --- | --- | --- | --- | --- | --- | --- | --- | --- |
| **Leonte UN** | - | 0.5456 (0.0149) | 0.0410 (0.0065) | 0.0225 (0.0044) | 0.0147 (0.0034) | 0.0010 (0.0010) | 0.0000 (0.0000) | 0.0010 (0.0010) | 0.0889 (0.0091) | 0.1670 (0.0097) |
| **Leonte BU** | 0.0003 | - | 0.0391 (0.0063) | 0.0635 (0.0090) | 0.0606 (0.0067) | 0.0186 (0.0036) | 0.0000 (0.0000) | 0.0010 (0.0010) | 0.0068 (0.0030) | 0.1084 (0.0097) |
| **Lindoso UN** | **0.0148** | **0.0192** | - | 0.0264 (0.0048) | 0.0010 (0.0010) | 0.0000 (0.0000) | 0.0000 (0.0000) | 0.0000 (0.0000) | 0.0098 (0.0029) | 0.0254 (0.0040) |
| **Lindoso BU** | **0.0184** | 0.0178 | **0.0219** | - | 0.0000 (0.0000) | 0.0000 (0.0000) | 0.0000 (0.0000) | 0.0000 (0.0000) | 0.0029 (0.0016) | 0.0293 (0.0050) |
| **S.Tirso UN** | **0.0179** | 0.0160 | **0.0392** | **0.0439** | - | 0.4981 (0.0160) | 0.0000 (0.0000) | 0.0000 (0.0000) | 0.0010 (0.0010) | 0.0166 (0.0039) |
| **S. Tirso BU** | **0.0256** | **0.0226** | **0.0413** | **0.0445** | 0.0013 | - | 0.0000 (0.0000) | 0.0000 (0.0000) | 0.0313 (0.0046) | 0.0166 (0.0042) |
| **Moledo UN** | **0.0522** | **0.0520** | **0.0735** | **0.0877** | **0.0456** | **0.0445** | - | 0.0010 (0.0010) | 0.0000 (0.0000) | 0.0000 (0.0000) |
| **Moledo BU** | **0.0315** | **0.0376** | **0.0657** | **0.0715** | **0.0401** | **0.0541** | **0.0467** | - | 0.0000 (0.0000) | 0.0000 (0.0000) |
| **P. Lanhoso UN** | 0.0098 | **0.0246** | **0.0227** | **0.0342** | **0.0360** | **0.0228** | **0.0770** | **0.0489** | - | 0.1416 (0.0119) |
| **P. Lanhoso BU** | 0.0089 | 0.0130 | **0.0218** | **0.0216** | **0.0224** | **0.0202** | **0.0744** | **0.0552** | 0.0105 | - |

**Table S5.** Pairwise genetic distance between all pairs of populations of *P. guadarramae*.

|  | **Leonte** | | **Lindoso** | | **S. Tirso** | | **Moledo** | | **P. Lanhoso** | |
| --- | --- | --- | --- | --- | --- | --- | --- | --- | --- | --- |
|  | UN | BU | UN | BU | UN | BU | UN | BU | UN | BU |
| Leonte UN | 0 | 0,0003 | 0,01498 | 0,01878 | 0,01819 | 0,02625 | 0,0551 | 0,0325 | 0,00994 | 0,00897 |
| Leonte BU | 0,0003 | 0 | 0,01958 | 0,01808 | 0,01628 | 0,02313 | 0,05481 | 0,03904 | 0,02526 | 0,01319 |
| Lindoso UN | 0,01498 | 0,01958 | 0 | 0,02243 | 0,04076 | 0,04306 | 0,07931 | 0,07036 | 0,02318 | 0,02232 |
| Lindoso BU | 0,01878 | 0,01808 | 0,02243 | 0 | 0,04588 | 0,04658 | 0,09616 | 0,07704 | 0,03542 | 0,02206 |
| S. Tirso UN | 0,01819 | 0,01628 | 0,04076 | 0,04588 | 0 | 0,00132 | 0,04781 | 0,04174 | 0,0373 | 0,02289 |
| S. Tirso BU | 0,02625 | 0,02313 | 0,04306 | 0,04658 | 0,00132 | 0 | 0,04655 | 0,05723 | 0,02337 | 0,02065 |
| Moledo UN | 0,0551 | 0,05481 | 0,07931 | 0,09616 | 0,04781 | 0,04655 | 0 | 0,04899 | 0,08337 | 0,08041 |
| Moledo BU | 0,0325 | 0,03904 | 0,07036 | 0,07704 | 0,04174 | 0,05723 | 0,04899 | 0 | 0,0514 | 0,05847 |
| P. Lanhoso UN | 0,00994 | 0,02526 | 0,02318 | 0,03542 | 0,0373 | 0,02337 | 0,08337 | 0,0514 | 0 | 0,01065 |
| P. Lanhoso BU | 0,00897 | 0,01319 | 0,02232 | 0,02206 | 0,02289 | 0,02065 | 0,08041 | 0,05847 | 0,01065 | 0 |

**Table S6.** Estimates of the critical M (Mc) value based on simulated equilibrium conditions under different parameter values. The number of loci and sample size were fixed to 6 and 20 diploid genotypes. “Mean size” refers to the mean size of large (i.e. non-SMM) mutations and %SMM refers to the proportion of stepwise mutations. The largest value obtained for this range of parameters is highlighted.

| **4Neµ** | **mean size** | **% SMM** | **critical M** |
| --- | --- | --- | --- |
| 20 | - | 1 | 0.743266 |
| 20 | 1.5 | 0.9 | 0.712872 |
| 20 | 3.5 | 0.9 | 0.588734 |
| 20 | 1.5 | 0.75 | 0.675783 |
| 20 | 3.5 | 0.75 | 0.500054 |
| 10 | - | 1 | 0.791667 |
| 10 | 1.5 | 0.9 | 0.759259 |
| 10 | 3.5 | 0.9 | 0.621204 |
| 10 | 1.5 | 0.75 | 0.718932 |
| 10 | 3.5 | 0.75 | 0.527392 |
| 5 | - | 1 | **0.8298** |
| 5 | 1.5 | 0.9 | 0.792163 |
| 5 | 3.5 | 0.9 | 0.654563 |
| 5 | 1.5 | 0.75 | 0.75463 |
| 5 | 3.5 | 0.75 | 0.549252 |

**Table S7.** Geographic coordinates and summary of the fire history of each population sampled. The time since the last fire (TSLF, in years) regards the Portuguese records (from 1975 to 2013). Therefore, the sites that have never been burnt since 1975 have the maximum possible time since the last fire of 39 years.

| **Population** | **Latitude** | **Longitude** | **Number of fires** | **TSLF** |
| --- | --- | --- | --- | --- |
| Leonte unburnt | 41.769500 | -8.149320 | 1 | 25 |
| Leonte burnt | 41.741320 | -8.172400 | 4 | 4 |
| Lindoso unburnt | 41.860000 | -8.220000 | 0 | 39 |
| Lindoso burnt | 41.875276 | -8.207062 | 2 | 4 |
| Santo Tirso unburnt | 41.313050 | -8.449233 | 3 | 12 |
| Santo Tirso burnt | 41.299290 | -8.432370 | 8 | 1 |
| Moledo unburnt | 41.838739 | -8.873442 | 0 | 39 |
| Moledo burnt | 41.829925 | -8.825788 | 7 | 1 |
| Póvoa de Lanhoso unburnt | 41.548425 | -8.140325 | 0 | 39 |
| Póvoa de Lanhoso burnt | 41.586216 | -8.281265 | 8 | 4 |

**Table S8.**  List of analysed microsatellite loci and respective characterization (Agostini et al., 2013) for *P. guadarramae*.

| **Locus** | **Repeat motif** | **Allele range (bp)** | **Fluorescent label** | **Multiplex** |
| --- | --- | --- | --- | --- |
| Ph17 | (TATC)_n_ | 149-225 | NED | C |
| Ph21 | (AGAT)_n_ | 127-189 | VIC |  |
| Ph30 | (TCTA)_n_ | 108-148 | FAM |  |
| Ph38 | (GATT)_n_ | 100-136 | PET |  |
| Ph50 | (ATGC)_n_ | 266-282 | VIC |  |
| Ph43 | (AGGG)_n_ | 136-153 | FAM | E |
| Ph70 | (CTT)_n_ | 162-195 | PET |  |
| Ph81 | (TGT)_n_ | 288-821 | FAM |  |
| Ph128 | (GTT)_n_ | 218-233 | VIC |  |
